# Improve Statistical Efficacy Inference: Global Reduction Rate in Disease Progression

**DOI:** 10.1002/alz.091799

**Published:** 2025-01-09

**Authors:** Guoqiao Wang, Kun Jin

**Affiliations:** ^1^ Washington University in St. Louis, School of Medicine, St. Louis, MO USA; ^2^ Anavex Life Sciences, New York, NY USA

## Abstract

**Background:**

In AD trials, the treatment effect is typically evaluated by estimating the absolute difference in change from baseline to the end‐of‐study visit (e.g., 18 months) between treatment arms using the MMRM model. To enhance clinical interpretability, this absolute difference is often converted into a percentage slowing relative to the placebo decline (e.g., 27% of CDR‐SB in lecanemab trial). We propose to model this percentage directly as a global reduction rate (GRR) in disease progression using multivariate proportional MMRM models (MpMMRM). MpMMRM estimates GRR across all multiple longitudinal and/or survival endpoints, either at the last study visit or for all post‐baseline visits. For example, donanemab lead to 35.9% reduction in iADRS, 36.0% in CDR‐SB, and 38.6% reduction in progression to more advanced disease stages as measured by CDR global, which can be model simultaneously to estimate a GRR. The objective of this presentation is to evaluate the advantages of utilizing GRR as an inference tool for treatment efficacy.

**Method:**

We will compare the global reduction rate (GRR) with three other statistical efficacy inference methods. These methods include: (i) Traditional MMRM inference using a single endpoint. (ii) Global statistics test (GST): GST combines z‐test statistics across multiple endpoints. (iii) Composite analysis utilizing a composite score derived from multiple endpoints. To evaluate these methods, we utilized simulated data that mimicked successful early Alzheimer’s disease trials.

**Result:**

Our preliminary simulation results showed that utilizing GRR across multiple longitudinal endpoints shows power advantages compared to the other methods, including GST. These advantages can be observed when considering the GRR at the last visit or overall post‐baseline visits. Additionally, estimating the GRR based on combined longitudinal and survival endpoints also demonstrates power advantages. Importantly, we also demonstrated that Type I errors are well‐controlled for all the methods considered in our simulations.

**Conclusion:**

GRR evaluates the totality of evidence of a treatment effect. It can be estimated using a one‐step approach based on well‐established SAS procedures. GRR does not require any additional assumptions and offers greater interpretability and power compared to traditional methods like GST and the composite method.

